# Exosome from indoleamine 2,3-dioxygenase-overexpressing bone marrow mesenchymal stem cells accelerates repair process of ischemia/reperfusion-induced acute kidney injury by regulating macrophages polarization

**DOI:** 10.1186/s13287-022-03075-9

**Published:** 2022-07-28

**Authors:** Xiangcheng Xie, Xiu Yang, Junxia Wu, Shengjie Tang, LiLi Yang, Xiao Fei, Ming Wang

**Affiliations:** 1 Department of Nephrology, Affiliated Hangzhou First People’s Hospital, Zhejiang University School of Medicine, No. 261, Huansha Road, Hangzhou, 310006 Zhejiang China; 2 Department of Nephrology, School of Medicine, The Second Affiliated Hospital, Zhejiang University, Hangzhou, China

**Keywords:** Acute kidney injury, Macrophage polarization, Mesenchymal stem cell-derived extracellular vesicles, Indoleamine 2,3-dioxygenase

## Abstract

**Background:**

Ischemia–reperfusion injury (IRI)-induced acute kidney injury (AKI) can repair itself completely. However, most moderate and severe patients undergoing IRI-AKI progress to chronic kidney disease due to incomplete repair. The present study is aimed to investigate the role of bone marrow mesenchymal stem cell-derived exosomes (MSC-Exo) with indoleamine 2,3-dioxygenase (IDO) overexpression on incomplete repair in mice after IRI.

**Methods:**

IRI mice was established by clamping the unilateral renal pedicles and challenged with MSC-Exo. Blood biochemical indexes and inflammation factors contents were measured by ELISA assay. Histopathological examinations were monitored by HE, Masson, Immunohistochemical and TUNEL staining. Immunofluorescence, flow cytometry and immunoblotting were used to detect the polarization of macrophages, respectively.

**Results:**

As compared to sham operation mice, IRI mice showed high contents of serum BUN and Scr, and more severe damaged kidney tissues on days 1 and 3, which all gradually declined over time, showing the lowest level on day 7 after injury. Once treated with MSCs-Exo that could directly transfer to kidney tubular cells, the restoration of kidney functions significantly accelerated by contrast to IRI mice, and the promotive effects were more obvious in IDO-overexpressed MSCs-Exo (MSCs-Exo-IDO)-treated IRI mice. Furthermore, MSCs-Exo-IDO administration also accelerated renal tubular cells proliferation, restrained tubular cells apoptosis, fibrosis and inflammation factor secretions during self-repair process compared to IRI mice, whose effects were higher than MSCs-Exo-NC-challenged IRI mice and IDO overexpressing plasmid-injected IRI mice. Mechanistically, MSCs-Exo-NC and MSCs-Exo-IDO exposure promoted the polarization from M1 macrophage to M2 macrophage, leading to more anti-inflammatory factors production, and subsequently altered the inflammatory microenvironment of renal tubular cells, which facilitated the self-repair process in mice after IRI.

**Conclusion:**

MSCs-derived exosome accelerated renal self-repair in IRI mice by activating M2 macrophages polarization, which effects were amplified by IDO overexpression in MSCs. Potentially, genetically modified MSCs-Exo is an effective approach to improve renal self-repair in IRI-AKI mice.

**Supplementary Information:**

The online version contains supplementary material available at 10.1186/s13287-022-03075-9.

## Introduction

Acute kidney injury (AKI) is one of the common and critical episodes in clinical settings, accounting for approximately 15% of hospitalized patients and 25% of ICU patients [1]. The rate of developing AKI in cardiac surgery patients is as high as 40% [2], the mortality rate of AKI patients is about 1.4%, and 1.7 million AKI patients die each year worldwide [3]. Although mild AKI might completely recover to normal kidney structure and function, emerging evidence shows that moderate and severe AKI patients can lead to incomplete repair of kidney structure and function, resulting in different degrees of chronic kidney disease (CKD) symptoms, which is called acute kidney injury-chronic kidney disease transformation [4]. Approximately 20–50% of AKI patients develop into CKD, and 3–15% patients develop into end-stage kidney disease (ESRD) [5]. AKI brings heavy economic burden to society and seriously affects the prognosis of patients. The long-term follow-up shows that AKI survivors have a significantly higher risk of death [6]. Therefore, it is of great clinical significance to explore effective methods to delay the transformation from AKI to CKD.

Ischemia–reperfusion injury (IRI) is the main cause of AKI in clinic which is characterized by endothelial cell activation, leukocyte recruitment and infiltration, death of tubular epithelial cells [7]. Severe ischemia, toxin and persistent inflammatory reaction can lead to capillary destruction, renal tubular loss, glomerulosclerosis and continuous vicious circle, leading to renal tubular atrophy and renal interstitial fibrosis, that is, “maladaptive repair,” which eventually resulting in CKD [8]. Among many factors implicated in the AKI pathogenesis, inflammation response runs through the whole process of IRI-AKI injury and self-repair, among which mononuclear phagocyte system (MoPh) is a key determinant involved in injury and repair [9]. Macrophages are an important part of innate immunity and play a central role in inflammation reaction and host defense [10]. Once induced by various factors, macrophages can differentiate into different phenotypes, that is, macrophage polarization. Macrophages are divided into M1-type macrophages and M2-type macrophages. M1 macrophage, also known as classic activated macrophage, has high expression of MHCII and costimulatory CD80/86, which can secrete pro-inflammatory factors and participate in pro-inflammatory reaction. M2 macrophage, also called alternative activated macrophage, is characterized by CD68-positive and CD163-positive, which can inhibit inflammation and promote the repair of injured tissue [11]. It is considered that the phenotype transformation of macrophages is an important indicator of AKI transition from inflammation injury stage to inflammation regression or even kidney injury repair stage. The ratio of M1 to M2 determines the progress of AKI towards kidney repair or CKD in some extend [12]. Hence, the polarization from M1 to M2 macrophage may contribute to the renal recovery after AKI. However, how to promote M1 to M2 polarization remains to be explored.

Studies have demonstrated that the therapeutic effect of stem cells mainly depends on its paracrine function to confine inflammation, regulate immune cells and activate endogenous repair pathways [13]. Exosome (Exo), a new carrier of intercellular signal transduction, participates in stem cell-mediated tissue repair through direct acting on target cells and the transfer of gene materials such as mRNA and miRNA to target cells [14]. However, its efficacy is limited and remains to be improved [13]. The genetically modified mesenchymal stem cells may be an effective method to boost the Evs efficacy [15, 16]. Indoleamine 2,3-dioxygenase (IDO) is composed of 403 amino acid residues, which is the only rate-limiting enzyme in cells except liver that can catalyze the epoxidation and cleavage of tryptophan indole and make it catabolize along kynurenine pathway (KP) [17]. Stem cells from IFN-*γ*-exposed amniotic fluid induce IDO-mediated immunoregulatory effect [18]. It is shown that IDO overexpression in bone marrow mesenchymal stem cell-secreted exosomes can down-regulate the expression of immune-promotive molecules in dendritic cells and up-regulate the number of Treg cells, producing immunosuppressive effect [19].

To date, it is unclear whether IDO is involved in EVs-mediated tissue repair and whether this effect is executed by affecting the macrophage phenotype remains to be clarified. Therefore, in this study we aim to testify that the overexpressed IDO in MSC-Exo promote renal repair after AKI by regulation of macrophage polarization.

## Materials and methods

### Cell separation and culture

4-week-old C57BL/6 mice were sacrificed by cervical dislocation after anesthetized with an intraperitoneal injection of 30 mg/kg pentobarbital sodium. The mouse was fixed on the plate with supine position, and the femur and tibia were taken under aseptic conditions and soaked in 75% alcohol for disinfection. After three times washing with PBS, the epiphyseal ends of femur and tibia were cut and the bone marrow cavity was exposed. Then the bone marrow was sucked using a 10 ml needle tube and placed into the culture medium containing 100μ/ml heparin, 100μ/ml penicillin and 100μ/ml streptomycin. The collected liquid contained bone marrow was mixed with 1.0 g/ml percoll, followed by 25 min density gradient centrifugation. Subsequently, the white layer at intermediate interface was collected and centrifugally cleaned twice with PBS solution containing double antibody. The isolates were inoculated into the coated culture bottle supplemented by L-DMEM culture medium containing 10% fetal bovine serum, 100u/ml penicillin, 100u/ml streptomycin and 5 μg/ml insulin and cultured in 5% CO_2_ environment at 37 °C. Raw 264.7 cells were cultured in high-glucose DMEM supplemented with 10% FBS, and 1% penicillin and streptomycin in a humidified incubator at 37 °C with 5% CO_2_. The culture medium was refreshed every day.

### Exosome isolation from MSCs with IDO overexpression

The control plasmid and IDO overexpression plasmid were constructed and transfected into MSCs cells, respectively. After 72 h, the supernatant of MSCs cells transfected with IDO overexpression plasmid and the supernatant of MSCs cells with control plasmid treatment were collected for exosomes extraction. MSCs supernatant was centrifuged at 2000 g for 30 min at 4 °C. Then, the supernatant was transferred to a new centrifuge tube, and centrifuged at 10,000 × g for 45 min at 4 °C to remove larger vesicles. Subsequently, the supernatant was filtered with a 0.45-μm filter membrane (Millipore, R6BA09493), and the filtrate was collected which was centrifuged again at 100,000 × g for 70 min at 4 °C in an ultracentrifuge (Hitachi, CP100MX). The supernatant was discarded, and the pellets were resuspended with 10 mL pre-cooled PBS. The pellets were centrifuged at 100,000 × g at 4 °C for 70 min. After discarding the supernatant, the pellets were resuspended in 100 μl precooled PBS. 10 μl particle resuspension was used to detect the particle size, 10 μL was used to extract total protein, and the left resuspension was stored at − 80 °C for the subsequent experiments.

### Transmission electron microscope (TEM)

The copper mesh was cleaned by plasma for 10–30 s, allowing the carbon side faces upwards. The copper mesh was put on the ice-cold sealing film, and subsequently 20 μL sample, 20 μL ultrapure water and 20 μL PTA dye solution were placed on the sealing film, respectively. After cooling, the carbon surface of the copper mesh was fastened on the sample droplets to absorb the sample for 1 min, and the excess liquid was sucked off by vertical contact between the filter paper and the copper mesh. Then, the carbon surface of copper mesh was attached to PTA droplets for 0.5–1 min dyeing. The copper mesh was placed on filter paper, allowing it to dry naturally in the shade, and the results were observed by electron microscope (FEI Tecnai G20 TWIN).

### Mouse model and treatment

6–8 week-old wild C57BL/6 mice were purchased from Beijing Weitong Lihua Experimental Animal Technology Co., Ltd. (Beijing, China). All mice were fed in a 12-h light/dark cycle room at a temperature of 24 ± 0.5 °C. All experimental protocols and procedures of this study were approved by Ethics Committee of Zhejiang Traditional Chinese Medical University and are in line with the National Institutes of Health's Guidelines for the Care and Use of Experimental Animals (8th Edition, 2010). The kidney IRI model was established using 6–8-week-old wild C57BL/6 mice. Firstly, the mice were intraperitoneally injected with 30 mg/kg pentobarbital (P-010, Sigma, USA). Secondly, the dorsal incision was made, and subsequently the unilateral renal pedicles (left side) were clamped for 25 min. In the sham operation group, the incision was made, but the renal pedicle was not clamped. Six hours after operation, mice received an intravenous injection of parental MSC-Exo, IDO-modified MSC-Exo (100 μg/mouse, once) or adenovirus containing IDO overexpression plasmid (2 × 10^9^/mouse). Then mice were fed for additional 1, 3, 7 or 28 days. After sacrificed, kidney tissues were harvested for paraffin embedding and western blotting. Spleen tissues were collected for mononuclear cells isolation.

### Scr and BUN measurements

Blood plasma was collected and centrifuged at 3500 rpm for 5 min. Serum creatinine (Scr) and blood urea nitrogen (BUN) in serum were determined using the corresponding detection kits (Scr: C011-1, Jiangcheng Bio, Nanjing; BUN: C013-2, Jiangcheng).

### Hematoxylin–eosin (HE) staining and immunohistochemistry (IHC)

Kidney tissues were fixed by 4% paraformaldehyde for more than 24 h, subsequently were embedded in paraffin. 5-μm paraffin slices were deparaffinized and immersed in xylene for 20 min, followed by a gradient of absolute ethanol. For HE staining, 5-μm thick slices were deparaffinized using xylene and hydrated and stained using HE reagent (Sigma, USA). Briefly, the slices were incubated with hematoxylin at room temperature for 5 min. After being washed, eosin was used to incubate the slices at room temperature for about 2 min. The kidney tissue morphology was observed under a microscope. For IHC staining, slices were incubated with 3% H_2_O_2_ for 5–10 min to remove endogenous peroxidase, followed with 5% BSA incubation for 1 h. Primary antibody against Ki67 (abcam, ab16667, 1: 200) was used to incubate the slice overnight at 4 °C. After the three times washing using PBS (3 min/each), the slices were incubated with the second antibody (Goat Anti-Rabbit IgG (H + L) HRP, affinity, S0001, 1: 200) at 37 °C for 30 min. Slices were rinsed with PBS for 4 times (3 min/each), and DAB color developing solution was added to each slice. Subsequently, the slices were re-dyed using Harris hematoxylin for about 2 min. After washing the slices in water and hyalinization with gradient alcohol, the slices were mounted using neutral gum. The staining results were observed under a microscope (OLYMPUS, BX53).

### TUNEL staining

Briefly, 5-μm paraffin slices were incubated with 20 μg/ml protease K, and subsequently fixed with 4% paraformaldehyde (pH7.4) solution for 5 min at room temperature. After three times washing using PBS, 100μL TdT enzyme reaction solution was added to the slices and allowed to incubate for 1 h in a wet box at 37 °C. Then the reaction was terminated using 2 × SSC solution for 15 min incubation at room temperature. After removing the endogenous catalase using 0.3% hydrogen peroxide (5 min incubation at room temperature), the slices were incubated with 100 µl Streptavidin HRP (streptavidin horseradish peroxidase) solution for 30 min at room temperature. Subsequently, 100 µl DAB color developing solution was added to the slices and the staining results were observed under the microscope. Consistently, the labeled slices were re-dyed by Mayer's hematoxylin for about 1 min and sealed with neutral gum. Air-dried slices can be captured under a microscope.

### Western blot

Total protein was extracted from kidney tissues using RIPA buffer. 40 μg protein was separated using sodium dodecyl sulfate polyacrylamide gel electrophoresis (SDS-PAGE). The gel was transferred to the activated-PVDF membrane. After the block of antigen using 5% skimmed milk powder for 2 h at room temperature, the primary antibodies (CD9, Affinity, Df6565, 1:1000; CD63, Affinity, Df2306, 1:1000; CD81, Affinity, Df2305, 1:1000; *α*-SMA, Affinity, AF1032, 1:1000; collagen I, Affinity, AF7001, 1:1000; collagen III, Affinity, AF0136, 1:1000; Arginase1, Affinity, Df6657, 1:1000; iNOS, Affinity, Af6270, 1:1000; caspase3, Abcam, Ab184787, 1:1000; GAPDH, abcam, ab9485, 1:1000) were used to incubate the immunoblots overnight at 4 °C. The next day, the membrane was washed for 5–6 times (5 min/each) with TBST. Subsequently, the blots were incubated with HRP labeled second antibody (Goat Anti-Rabbit IgG (H + L) HRP, affinity, S0001, 1: 5000) in a shaker at 37 °C for 2 h. The immunoblots were visualized by adding ECL reagent and photographed by Gel imager. The results were analyzed by Image J software (National Institutes of Health, USA).

### Masson staining

The tissue samples were embedded in paraffin wax. 5-μm-thick tissues were placed on separate glass slides and subsequently deparaffinized and immersed in xylene for 20 min. After hematoxylin–eosin (HE) staining, the slices were stained with Masson staining solution. The severity of tubulointerstitial damage was graded according to interstitial collagen deposition using Masson's trichrome staining.

### Immunofluorescence

In brief, paraffin sections of kidney tissues were dewaxed and rehydrated. The slices were subjected to immunofluorescence staining with the primary antibodies against F4/80 (ab6640, Abcam; diluted 1:100), CD206 monoclonal (DF4149, Affinity; diluted 1:100) or HLA-DR (MA5-32,232, Invitrogen; diluted 1:100) at 4 °C overnight. The second day, the slices were washed with 1 × PBS for 5 min/3 times and incubated with corresponding secondary antibody (Molecular Probes, USA; diluted 1:500) for 1 h at room temperature. Dihydrochloride (DAPI, Beyotime, China; diluted 1:300) was used to display the nucleus. The detection of F4/80, HLA-DR and CD206 was performed under a fluorescence microscope (IX71, Olympus, Japan). The positive cells were counted using Image pro plus software.

### ELISA

The concentrations of IL-1*β* (PI301, Beyotime), IL-6 (PI326, Beyotime), TNF-*α* (PT512, Beyotime) and IL-10 (PI523, Beyotime) in kidney tissues or macrophages were measured by enzyme-linked immunosorbent assay (ELISA) kits according to the manufacturer’s protocols. The OD values were read using a Victor 3 multilabel plate reader (PerkinElmer).

### Flow cytometry

Cells were digested with 0.25% trypsin without EDTA. Cell pellets were collected after 5 min centrifugation at 1500 rpm. After resuspending the pellets using PBS, the corresponding antibodies (HLA-DR, Invitrogen, MA5-16,502; CD206, Invitrogen, MA5-16,870) were added to the cell resuspension for 30 min incubation at 4 °C. After 5 min centrifugation at 1500 rpm, the pellets were resuspended with 200 μl PBS, and flow cytometry (Beckmancoulter, cytoFLEX) was used for the subsequent detection.

### Statistical analysis

All data are expressed as the mean ± standard deviation (SD). One-way analysis of variance (ANOVA) followed by a post hoc Student–Newman–Keuls multiple comparisons test was used to evaluate the differences between groups. All statistical values were calculated by SPSS 22.0 (V22.0, SPSS, Inc., IL, USA). *P* < 0.05 was defined as statistically significant.

## Results

### MSC-Exo-IDO accelerates the renal function repair in renal ischemia–reperfusion mice

**Fig. 1:**
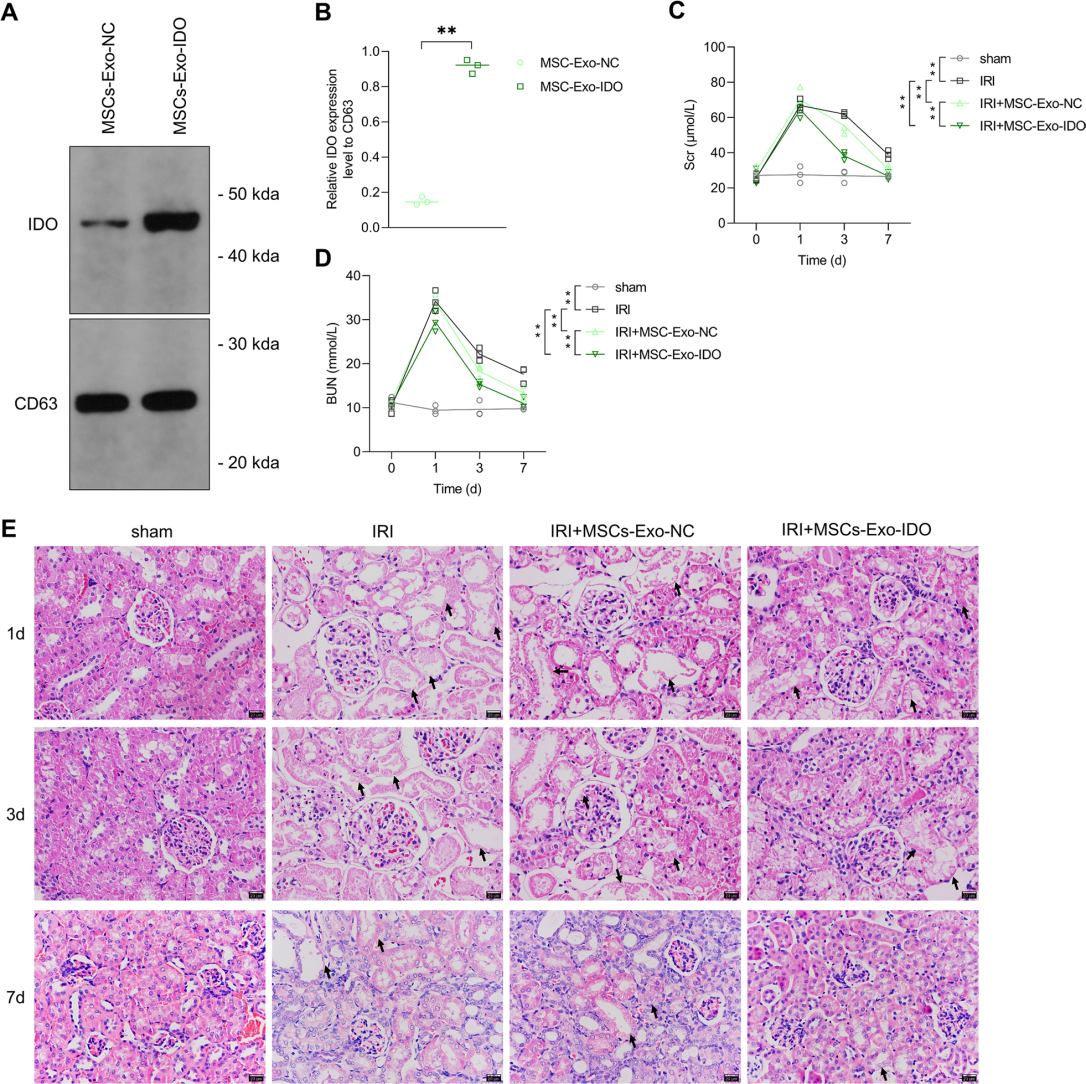
Effect of MSCs-Exo-IDO on renal functions in IRI mouse model. **A** The protein content of IDO in MSCs-Exo-NC or MSCs-Exo-IDO was firstly detected by Western Blot (*n* = 3). **B** Quantization diagram of panel A as shown in the right. Then, IRI mouse model was established. IRI mice were challenged with MSCs-Exo-NC or MSCs-Exo-IDO. Next, serum was obtained from different group mice on days 0, 1, 3 and 7 after IRI. BUN **C** and Scr **D** contents were detected by the corresponding ELISA kits (*n* = 3/group, the results for the same mice at each time). **E** Kidney tissues were collected from the four groups on day 1, 3 and 7 after IRI (*n* = 3/group at each time). Kidney tissues in different groups were obtained and histopathology was monitored by HE staining. Arrowheads indicated the damaged tubular. The line chart is presented by Means ± SD, and the statistical analysis was performed using one-way ANOVA. **P < 0.01

To investigate the role of MSCs-Exo or IDO-overexpressed MSCs-Exo (MSCs-Exo-IDO) on renal self-repair after ischemia–reperfusion (IRI), MSCs were isolated from mouse bone marrow. Subsequently, MSCs exosomes were also isolated using density gradient centrifugation method. As shown in Additional file 1: Fig. S1A, the isolated particles expressed high levels of CD63, CD9 and CD81 that were the exosomal biomarkers (Additional file 1: Fig. S1A). Additionally, the TEM results showed that particles were round and its diameter ranged from 40 to 100 nm (Additional file 1: Fig. S1B). Therefore, MSCs-derived exosomes were successfully extracted. To further prove that MSCs-secreted exosome could directly transfer to damaged kidney, the isolated exosomes were labeled by DiD before injection. As shown in Additional file 2: Fig. S2, a lot of fluorescence intensity of DiD was observed in tubular of kidney tissues obtained from IRI mice. However, no fluorescence of DiD was found in PBS-injected mice (Additional file 2: Fig. S2). The data suggested that MSCs-derived exosome can transfer to kidney tubular to function as an important cellular regulator. In addition to that, more protein level of IDO was observed in IDO-overexpressed MSCs-Exo as compared to that in MSCs-Exo (Fig. 1A, B), which suggested that IDO-overexpressed MSCs-Exo was also successfully established. Then, acute IRI mice were established by clamping the unilateral renal pedicles. As shown in Fig. 1C and D, compared with sham operation group, BUN and Scr contents increased by 2.5-fold in IRI mouse model group treated with or without MSCs-Exo (Day 1). By contrast, BUN and Scr levels decreased significantly on the third day after IRI, which declined gradually along with time increase, showing the lowest on day 7 after IRI in the three model groups compared to sham operation group. The data indicated that IRI mouse kidney model was successfully constructed. However, compared with IRI model mice without Exo treatment, the rates of BUN and Scr decrease were faster (the 3rd and 7th day after IRI) in mice treated with MSC-Exo treatment. Once IDO was overexpressed in MSCs, the MSCs-Exo-IDO challenge further accelerated the speed reduction of BUN and Scr on day 3 and 7 after IRI, as compared with IRI mice treated without or with parental MSCs-Exo-NC (Fig. 1C, D). Actually, MSCs-Exo-IDO-administrated mice presented the same contents of BUN and Scr by contrast with the sham operation group on day 7. On days 1 and 3 after IRI, HE staining revealed that there was severe kidney injury with tubular damage in IRI mice compared to those in sham surgery group (Fig. 1E). The administration of MSCs-Exo or MSCs-Exo-IDO significantly reduced the impaired symptoms in kidney tissues, and there was a higher repair effect in MSCs-Exo-IDO-exposed mice than that in MSCs-Exo-exposed mice on day 7 after IRI (Fig. 1E). There were no differences in kidney injury among these groups on day 1 after IRI (Fig. 1E). Collectively, MSCs-Exo promotes the renal repair process in mice after IRI and IDO-overexpressed MSCs-Exo plays a key determinant during the repair progression.

### MSC-Exo-IDO alleviates renal tubular cell apoptosis in renal IRI mice

**Fig. 2:**
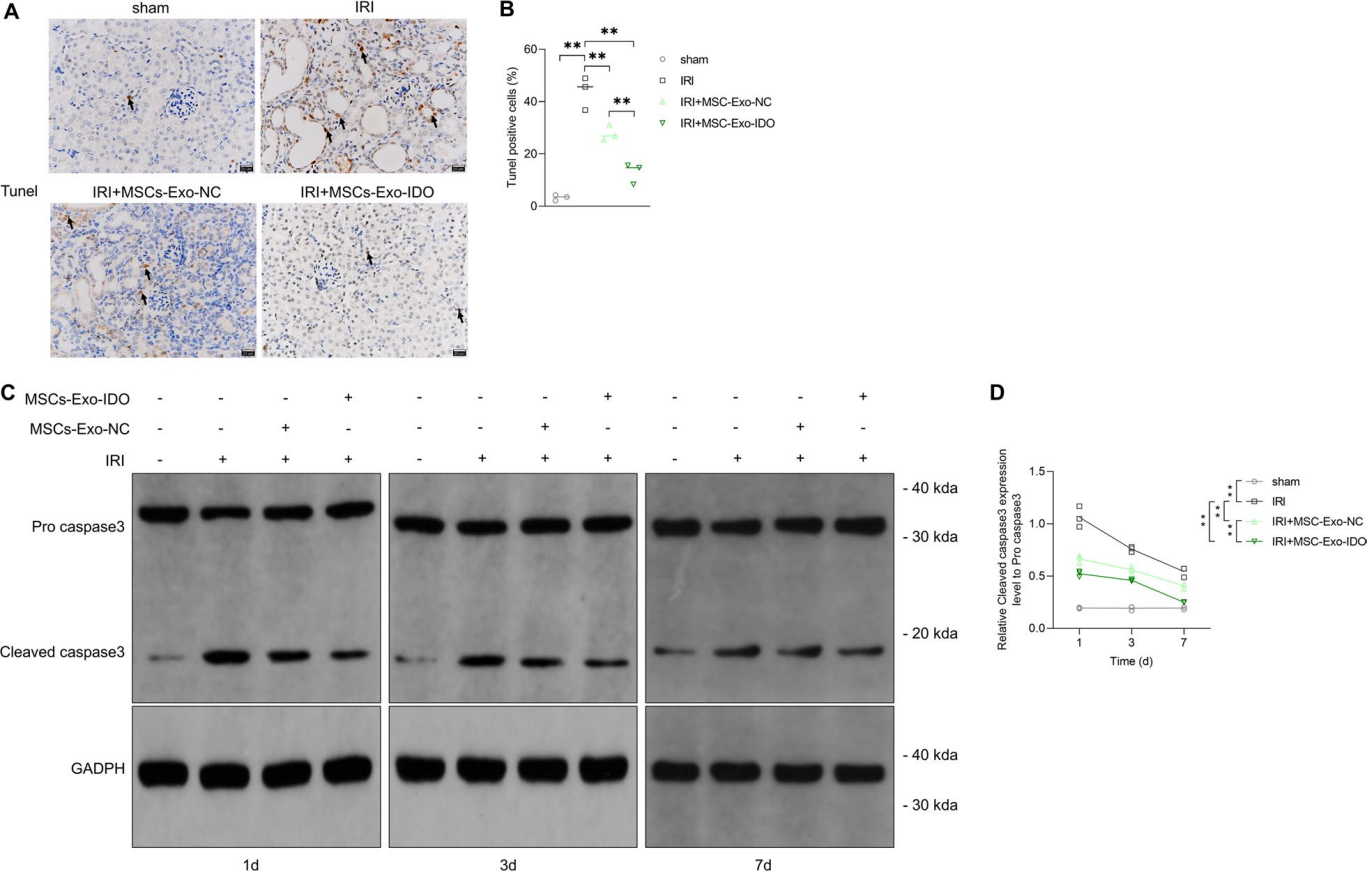
Role of MSCs-Exo-IDO on renal tubular cell apoptosis in IRI mice. **A** Kidney tissues were collected from the four groups on day 3 after IRI (*n* = 3/group). Apoptotic cells were determined by TUNEL staining assay, and cell-labeled brown color were considered as the apoptotic renal tubular cells. Arrowheads indicated the TUNEL-positive cells. **B** Quantization diagram of TUNEL signal-positive cells as shown in the right. **C** Protein levels of pro-caspase3 and cleaved caspase3 were detected by WB assay in kidney tissues collected from day1, 3 and 7 after IRI (*n* = 3/group at each time). **D** Quantization diagram of cleaved caspase3 as shown in the right. Means ± SD was used for histogram presentation. **P < 0.01

Next, apoptotic events in renal tubular cells were evaluated in our study. As expected, there were more apoptotic cells in IRI mouse kidney compared with sham operation group (Fig. 2A, the left two images). However, TUNEL positive signals (apoptotic cells) notably decreased in MSCs-Exo-NC or MSCs-Exo-IDO treated mouse kidney by contrast to that in IRI mouse kidney (Fig. 2A, the right two images versus the second image), showing a 1.6-fold decrease in MSCs-Exo-NC-challenged mice and 3.6-fold decrease in MSCs-Exo-IDO-challenged mice (Fig. 2B). Consistently, in kidney tissues of IRI mice, the expression of cleaved caspase3 in kidney tissue of model group increased on day3 1 and 3 after IRI compared with the control group, while the expression of pro caspase3 decreased with time increase, only showing moderately higher on day 7 than the control group (Fig. 2C, D). Compared with IRI group, MSCs-Exo-NC can inhibit the expression of cleaved caspase3, and the inhibitory effect of MSCs-Exo-IDO is stronger than that of MSCs-Exo-NC from day 1 to day 7 (Fig. 2C, D). It is suggested that MSC-Exo can alleviate the renal tubular cell apoptosis of IRI mouse kidney model, and IDO-overexpressed MSCs-Exo has stronger therapeutic effect.

### MSC-Exo-IDO accelerates the renal tubular cells proliferation in renal IRI mice

**Fig. 3:**
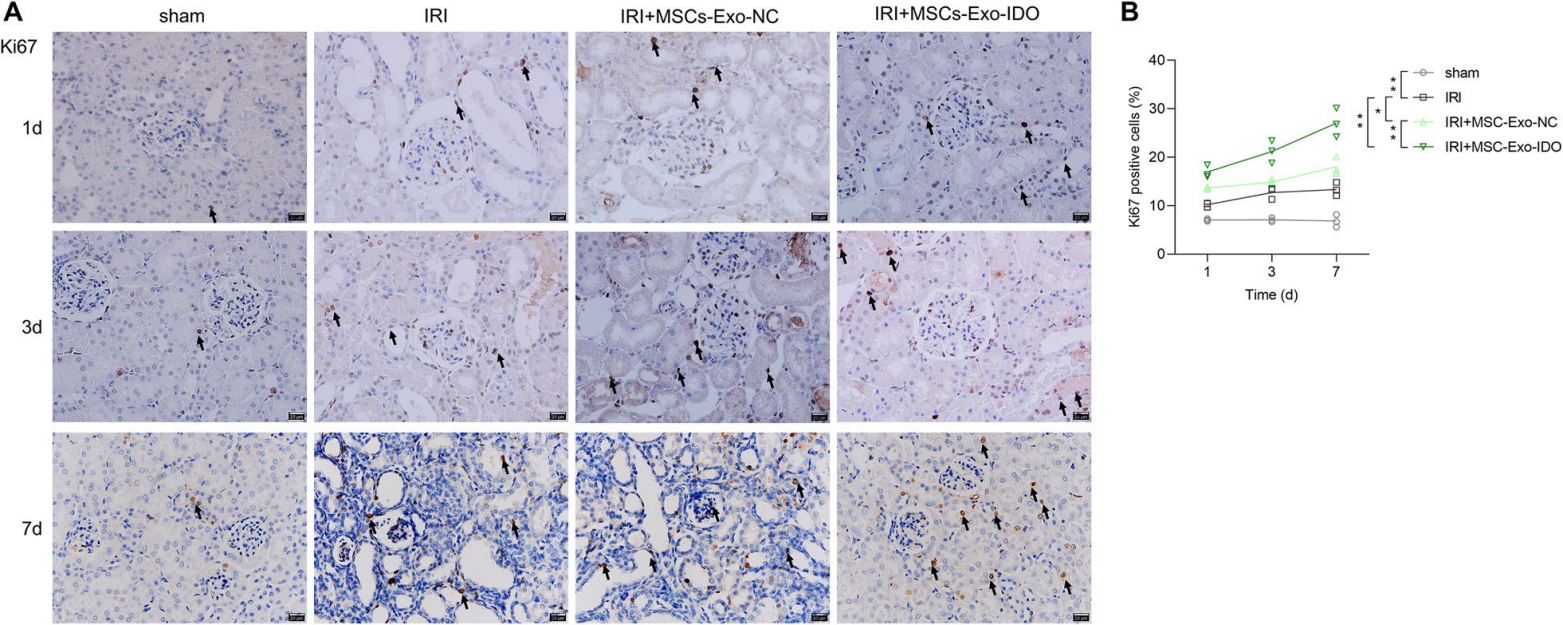
Effect of MSCs-Exo-IDO on renal tubular cell proliferation in IRI mouse model. **A** On day 1, 3 and 7 after IRI, kidney tissues were collected from sham operation group, IRI model mice, MSCs-Exo-NC-treated IRI mice and MSCs-Exo-IDO-treated IRI mice (*n* = 3/group at each time). Subsequently, IHC staining was used to detect Ki67 expression and the rate of Ki67-positive cells in kidney tissues. Arrowheads indicated the ki67-positive cells. Scale bar: 20 μm. **B** Quantization diagram of panel A. The histogram is presented by Means ± SD. *P < 0.05; **P < 0.01

At days 1 and 3 of modeling, compared with sham operation group, Ki67 positive cell rate significantly increased by 1.5-fold and 2.5-fold in renal ischemia–reperfusion (IRI) mouse kidney model group (Fig. 3, *P* < 0.01). Once exposed with bone marrow mesenchymal stem cell-derived exosomes (MSCs-Exo), Ki67 positive cell rate increased again by 1.3-fold on day 1, 1.1-fold on day 3 and 1.3-fold on day 7 as compared to that in IRI model mice (Fig. 3, *P* < 0.05). Of note, the challenge of exosomes isolated from IDO-overexpressed MSCs further raised the Ki67 positive cell rate by 1.5-fold on day 7 compared to parental MSCs-Exo-exposed mice kidney (*P* < 0.01), and increased by 2.1-fold on day 7 as compared with IRI model mice (*P* < 0.01) (Fig. 3). Therefore, MSCs-Exo can promote the proliferation of renal tubular cells during self-repair process after IRI, and IDO overexpressed MSCs-Exo plays a promotive effect on the repair of renal tubular in Exo-treated IRI mice.

### MSC-Exo-IDO limits renal fibrosis process in mice after IRI

**Fig. 4:**
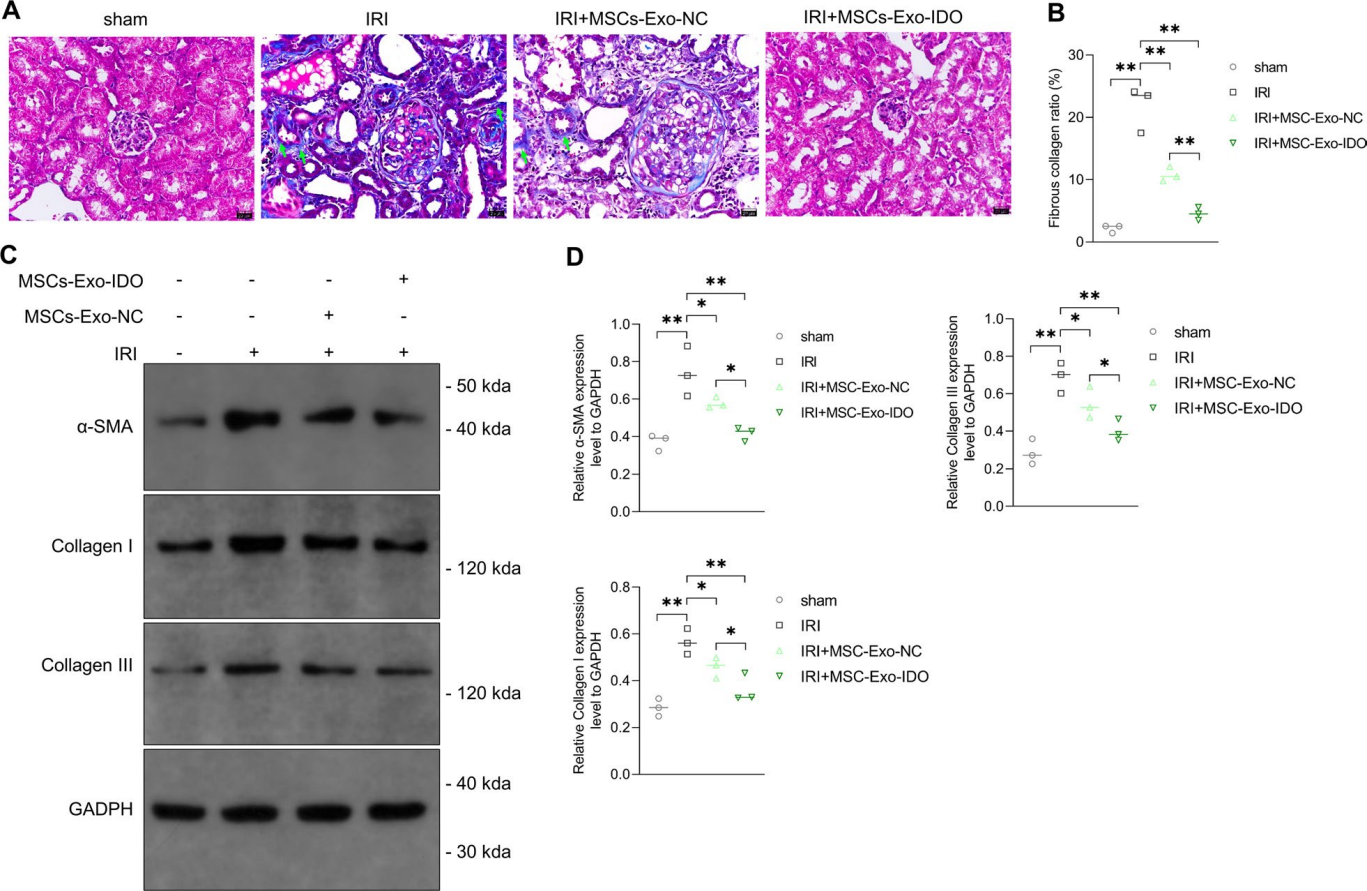
Effect of MSCs-Exo-IDO on renal fibrosis in IRI mouse model. **A** Kidney tissues were harvested from the four groups on day 28 after IRI (*n* = 3/group). Kidney tissues in different groups were obtained and histopathology was monitored by Masson staining. Arrowheads indicated the collagen deposition and fibrotic lesions. **B** Quantization diagram of panel A as shown in the right. **C** Western Blot was used to detect the expression of *α*-SMA, collagen I and collagen III in the above kidney tissues (*n* = 3/group). **D** Quantization diagram of panel C as shown in the right. The histogram is presented by Means ± SD. *P < 0.05; **P < 0.01

IRI model always was accompanied by the fibrosis information in kidney. Thus, the fibrosis process and fibrosis-related proteins were also assessed in our research. Masson’s staining results indicated that IRI model mice clearly presented more blue collagen deposition in kidney tissues by contrast with Sham control mice. In comparison with the model group, MSCs-Exo administration notably reduced the collagen deposition and fibrotic lesion, which showed the strongest inhibitory effect in IDO-overexpressed MSCs-Exo-challenged IRI mice (Fig. 4A, B). In IRI mice kidney (Day 2), *α*-SMA, collagen I and collagen III protein levels obviously elevated as compared with the sham operation group (Fig. 4C, D). Along with the exposure of MSCs-Exo, the expression of *α*-SMA, collagen I and collagen III protein declined sharply by 1.4-fold in MSCs-Exo-NC-treated mice and by nearly twofold in MSCs-Exo-IDO-treated group (Fig. 4C, D). The results indicated that MSCs-Exo could alleviate the process of renal fibrosis in IRI mouse kidney model, and IDO of MSCs-Exo possibly served as a critical suppressor during this process.

### MSC-Exo-IDO can alleviate kidney inflammation and M1 macrophages infiltration induced by kidney ischemia–reperfusion in mice

**Fig. 5:**
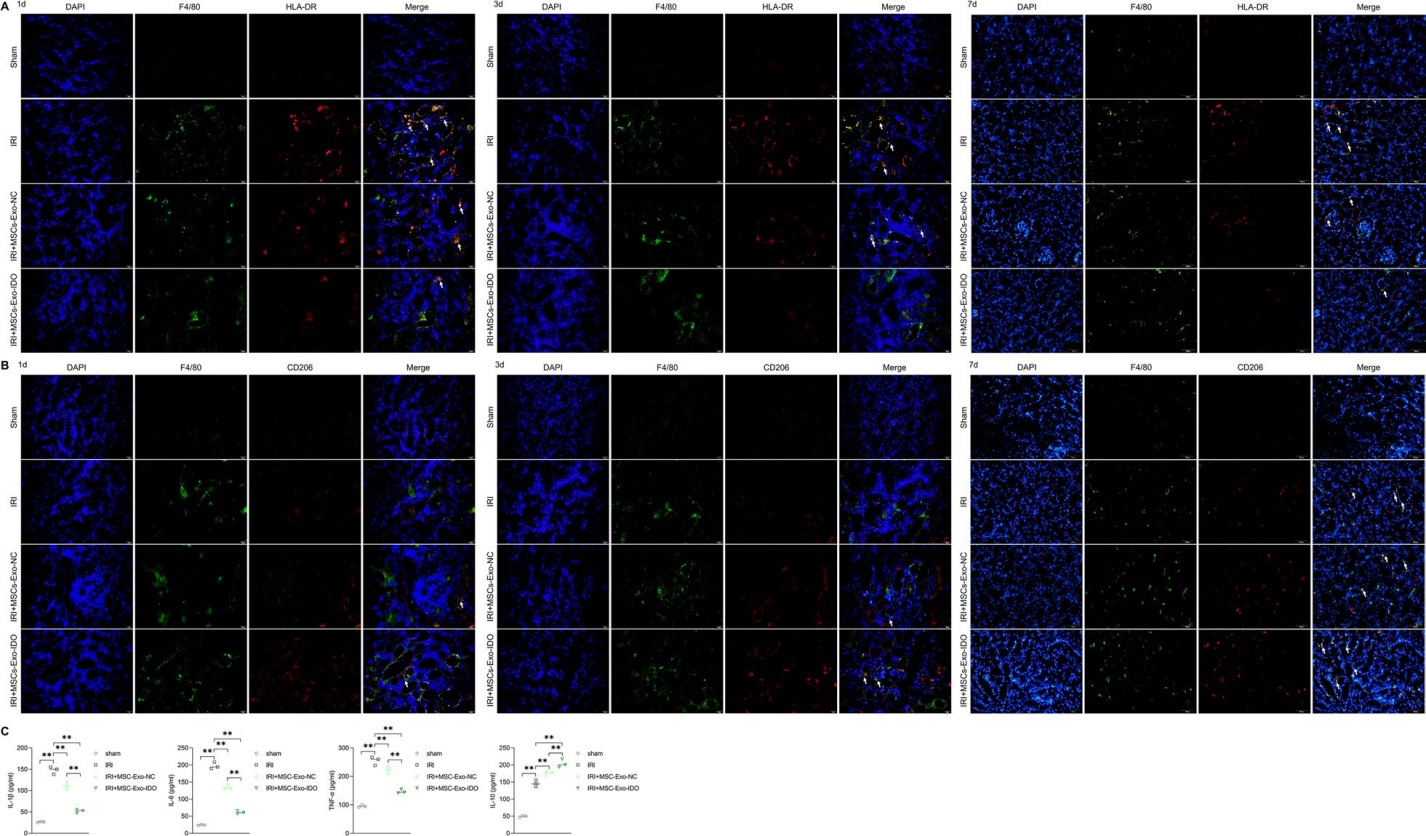
Effect of MSCs-Exo-IDO on macrophage infiltration and inflammation in kidney from IRI mice. **A** Kidney tissues were collected from the four groups on day 1, 3 and 7 after IRI (*n* = 3/group at each time). M1 macrophages in kidney tissues were determined by IF assay and cell-labeled yellow color were considered as M1 macrophage. **B** M2 macrophage in the above kidney tissues were determined by IF assay, and cell-labeled yellow color were considered as M2 macrophage (*n* = 3/group at each time). **C** The content of IL-1*β*, IL-6, TNF-α and IL-10 in kidney tissue of IRI mice was detected by ELISA (*n* = 3/group). The histogram is presented by Means ± SD. *P < 0.05; **P < 0.01

Inflammation response plays a switch role on the formation and self-repair processes of IRI. Thus, IF staining was applied to determine the macrophages infiltration in different mice tissues. As shown in Fig. 5A, B, more M1-type macrophages (F4/80^+^/HLA-DR^+^) populations and M2-type macrophages (F4/80^+^/CD206^+^) populations were appeared in IRI mice kidney tissues on days 1 and 3 compared to that in Sham control mice (Fig. 5A, B). MSCs-Exo exposure reduced the populations of infiltrated M1 macrophages, whereas induced the increase of infiltrated M2 macrophages, which effects were enhanced once treated with IDO-overexpressed MSCs-Exo on day 3 (Fig. 5A, B). No differences among these were observed on day 1 after IRI (Fig. 5A, B). As shown in Fig. 5C, several pro-inflammation or anti-inflammation factors were measured to assess the alteration of inflammation reaction. Compared with sham operation group, the contents of pro-inflammatory IL-1*β*, IL-6 and TNF-*α* notably increased in IRI mouse kidney model group. Once treated with MSCs-Exo, the contents of IL-1*β*, IL-6 and TNF-*α* decreased significantly compared to the IRI mice, and there was a sharply decline in MSCs-Exo-IDO-treated mice, showing the highest decrease rate compared to MSCs-Exo-NC-exposed group (Fig. 5C). Accompanied by the process of self-repair, the anti-inflammation factor IL-10 also increased in IRI mice by contrast to the control group (Fig. 5C). However, the content of IL-10 increased further when mice were exposed with MSC-Exo-NC or MSC-Exo-IDO. And it presented a stronger promotive effect on the expression of IL-10 in MSC-Exo-IDO-exposed mice than that in MSC-Exo-NC-exposed mice (Fig. 5C). Thus, MSCs-Exo alleviates the secretion of inflammation factors of IRI mouse kidney model. Possibly, IDO overexpression had a promotive role on MSCs-Exo-mediated anti-inflammation effect.

### MSC-Exo promotes the polarization from M1 macrophage to M2 macrophage

**Fig. 6:**
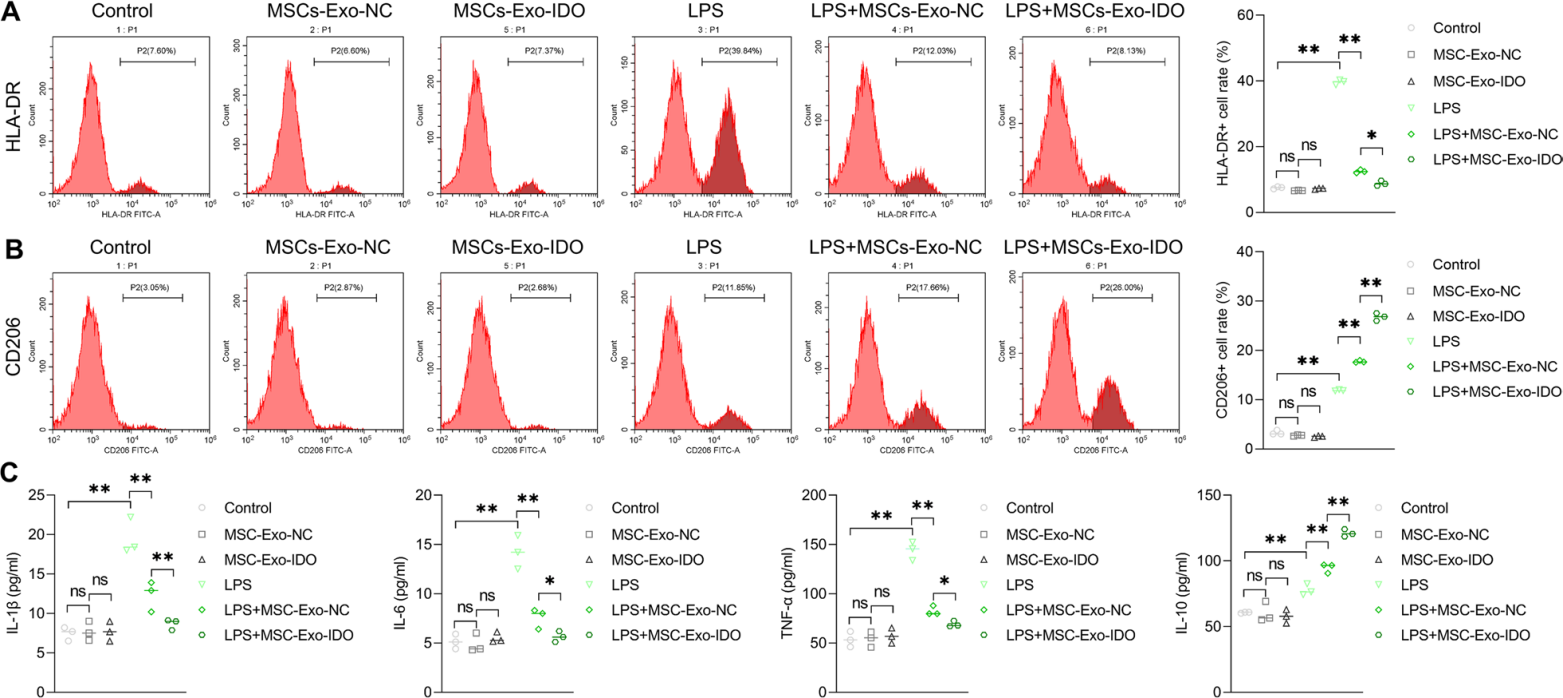
Effect of MSCs-Exo-IDO on macrophage polarization. HLA-DR-positive **A** and CD206-positive **B** cell population in LPS-induced RAW264.7 cells treated with or without MSCs-Exo were detected by flow cytometry (*n* = 3/group). Quantization diagram as shown in the right. **C** ELISA was used to detect the secretion of inflammatory factors IL-1*β*, IL-6, TNF-*α* and IL-10 in LPS-exposed RAW264.7 cells with or without MSCs-Exo. Mean ± SD was used for histogram presentation (*n* = 3/group). **P < 0.01

Subsequently, macrophages were employed to explore the inflammation regulatory mechanism during self-repair process in IRI mice treated with or without MSCs-Exo in vitro. The polarization rate of macrophage was detected by flow cytometry. As shown in Fig. 6, MSCs-Exo administration did not affect the polarization of resting macrophage without LPS treatment (no effect on the HLA-DR- or CD206-positive cell rates) and also could not change the content of inflammatory factors from M1 macrophage and M2 macrophage. LPS treatment significantly promoted the HLA-DR-positive cell (M1 macrophage marker) rate and the CD206-positive cell rate (M2 macrophage marker) and enhanced the secretion of proinflammatory factor IL-1*β*, IL-6, TNF-*α* (from M1 macrophage) and anti-inflammatory factor IL-10 (from M2 macrophage) resulting in macrophage polarization (Fig. 6A–C). By contrast, MSC-Exo administration notably inhibited LPS-induced macrophage M1 polarization (HLA-DR-positive cell rate decreased sharply) and the secretion of IL-1*β*, IL-6 and TNF-*α*, whereas promoted its transformation to M2 type (CD206-positive cell rate moderately increased) and the secretion of anti-inflammatory factor IL-10 (Fig. 6A–C). Consistent with the above finding, LPS notably increased the expression of iNOS (M1 macrophage marker) and Arginase1 (M2 macrophage marker), suggesting that LPS treatment induce the activation of macrophage indeed (Additional file 3: Fig. S3). MSC-Exo administration inhibited LPS-induced expression of iNOS, while promoted the expression of Arginase 1. Of note, IDO-overexpressed MSCs-Exo treatment had a stronger effect on reducing M1 polarization and promoting M2 polarization, leading to stronger anti-inflammatory effects (Fig. 6A–C). Therefore, it is suggested that MSCs-Exo promote the transformation of M1 type macrophages to M2 type macrophages and promote the M2-macrophage-mediated anti-inflammation response. Possibly, IDO overexpression had a promotive role on MSCs-Exo-mediated macrophage polarization and enhanced the anti-inflammation effect.

In addition to that, we also compared the therapeutic effect of IDO-targeted therapy to the therapeutic effect of exosomal IDO on IR-induced AKI model. The data proved that IDO-overexpressing MSCs-derived exosome harbored a stronger therapeutic effect on kidney damage than adenovirus mediated IDO overexpression (Additional file 4: Fig. S4). BUN and Scr levels and kidney injury significantly decreased from day 1 to day 7, which had a faster reduction rate in exosomal IDO-challenged group than IDO-targeted therapy group (Additional file 4: Fig. S4A–C). Of note, IDO-targeted therapy presented more IDO expression in kidney tissues than that in exosomal IDO-exposed group (Additional file 4: Fig. S4D, E). The difference in therapeutic effect between the two groups was possibly due to that exosome could carry more effective IDO to the target cells (kidney tubular cells) than adenovirus vector.

## Discussion

Ischemia–reperfusion (IR) injury is the main cause of AKI [20, 21]. Renal IRI is associated with high rates of mortality and end-stage kidney failure, and notably, even if patients recover from initial injury, renal IRI may have lasting effects including the development of CKD [22]. Macrophages play a pivotal role in kidney injury and self-repair. In the early stage of ischemia–reperfusion injury (within 48 h), M1 type macrophages appears in kidney tissue, while M2 type macrophages dominates in the later stage [23]. Eliminating macrophages before ischemia–reperfusion can alleviate kidney injury, while eliminating macrophages after 3⁓5d days of injury can slow down the proliferation and repair ability of renal tubular cells [23]. The transformation of macrophages from M1 type to M2 type can produce a large number of growth factors, such as platelet-derived growth factor (PDGF), transforming growth factor beta 1 (TGF-*β*1), insulin-like growth factor I (IGF-1) and vascular endothelial growth factor A (VEGF-A) [24]. IGF-1 and VEGF-A promote the regeneration and repair of renal tubular epithelial cells [25]. Therefore, macrophages are the key factor to regulate the self-repair after AKI. It is considered that the phenotype transformation of macrophages is an important sign of AKI's transition from inflammation injury stage to inflammation regression or kidney injury repair stage. Therefore, the advantaged ratio between M1 and M2 determines AKI's self-repair progress [26]. In the present study, we found that that MSCs-Exo promoted the transformation of M1 type macrophages to M2 type macrophages, and IDO-overexpressed MSCs-Exo had a higher efficacy to inhibit the process of renal fibrosis and apoptosis in IRI mouse kidney model, accelerating the self-repair process in mice after IRI. Our findings revealed the anti-inflammation mechanism of MSCs-Exo in mice after IRI and MSCs-Exo-mediated the acceleration role on renal self-repair possibly contributed to the therapy of IRI, probably depending on macrophage polarization.

Mesenchymal stem cell (MSC) is a kind of stem cell which originated from mesoderm and has the potential of self-stem cell renewal and multi-directional differentiation. MSCs mainly exist in bone marrow and also exist in fat, muscle, skin, umbilical cord, placenta and other tissues. Stimulated by different incentives, MSCs can differentiate into mesoderm cells such as osteocytes, chondrocytes and adipocytes and can also differentiate into ectoderm nerve cells, glial cells and endoderm liver oval cells [27]. MSCs-derived extracellular vesicles can release many types of vesicles, which are mainly divided into exosomes, microvesicles and apoptotic bodies according to their size and source [28]. Accumulating evidences have confirmed that exosome participate in stem cell repair via regulating immunomodulatory functions. MSCs-derived exosomes carrying genetic and protein material transfer to recipient cells, activating several repair mechanisms to ameliorate renal injury [29]. MSCs-Exo can induce the reprogramming of injured cells and renal tubular cell proliferation and inhibit cell apoptosis and inflammation in different kidney injury models [30]. Additionally, MSC-Exo has become an important alternative therapy of MSCs-based disease therapy. As low-risk cell-free MSC-based therapies, MSCs-Exo-based therapies have important therapeutic potential and application potential in the treatment of multiple diseases. In this study, MSCs-Exo administration relived the kidney injury and accelerated the self-repair process through inhibiting fibrosis and apoptosis and promoting cell proliferation in mice after IRI. The finding was consistent with the previous studies, which indicated that MSCs-Exo was highly likely to have therapeutic and application potential in acute renal IRI injury. Actually, exosomes can promote macrophage transformation from M1 type to M2 type in the mouse myocardial cell injury model, achieving the effect of myocardial cell injury repair [31]. Furthermore, our data proved that the improvement role on kidney injury mediated by MSCs-Exo was possibly through promoting the polarization from M1 macrophage to M2 macrophage and anti-inflammation role of M2 macrophage. Thus, immunomodulatory functions of MSCs-Exo implicated in the self-repair process in mice after IRI.

Accumulating studies have confirmed that exosomes can produce anti-inflammatory and exert tissue repair effects by regulating macrophage polarization, but it is not clear that how exosomes regulates macrophage polarization to repair renal tubular epithelial cells after renal IRI. Exosomes secreted by most tissues and cells will be released into blood and body fluids and exosomes can carry protein, lipid, DNA, mRNA and microRNAs to target cells, regulating multi-cellular behaviors [32]. Exosomes mainly works in three ways: Firstly, it fuses with target cells or directly releases their contents to target cells; secondly, exosomes combine with cell surface receptor to activate target cells; thirdly, exosomal contents were transmitted to target cells by endocytosis [14]. Actually, MSCs-Exo-mediated regulatory function mainly depends on the biomaterial transmit from exosomes to the target cells. MSCs-Exo regulates macrophage polarization to promote cartilage repair by carrying the exosomal CD73 to the target cells [33]. Melatonin-stimulated normal MSCs-Exo can carry the miR-4516 to the MSCs isolated from CKD patients, subsequently activates regenerative potential of chronic kidney disease-derived mesenchymal stem/stromal cells [34]. Besides, the alteration of exosomal contents such as RNA or protein can improve its therapeutic potential. miR-214 inhibitor-transfected HEK293T can produce anti-miR-214-enriched exosomes, which subsequently transmit the miR-214 to gastric cancer cells, promoting the radiosensitivity of gastric cancer cells [35]. miR-92a-3p-overexpressed MSCs-Exo can regulate chondrogenesis and cartilage homeostasis via carrying the miR-92a-3p to the target cells [36]. Previous studies show that dendritic cells (DCs) are the main source cell of IDO, and IFN-*γ* can activate the non-classical pathway of NF-κB under the stimulation of IKK*α* in DCs, resulting in the transcriptional expression of IDO [37, 38]. In the present study, IDO protein was existed in MSCs-Exo, and IDO-overexpressed MSCs-Exo contained more IDO protein. Of note, IDO-upregulated MSCs-Exo had a stronger promotive effect on renal tissue repair. IDO-overexpressed MSCs-derived exosomes significantly relived kidney damage, accelerated renal self-repair process in mice after IRI, and the improvement role was notably higher than that in MSCs-Exo-challenged mice. The data indicated that IDO carried by exosomes possibly was the determinant factor for MSCs-Exo-IDO-mediated self-repair. Therefore, IDO-overexpressed MSCs can produce more IDO-enriched exosomes, which subsequently accelerate the renal repair function of MSCs-Exo.

IDO can recognize indole-containing substrates, which are mainly composed of endothelial cells, fibroblasts, bone marrow-derived suppressor cells, DCs and macrophages, and the expression level is very low under physiological conditions [39]. Tryptophan breakdown via IDO and neopterin production by GTP-cyclohydrolase-I are initiated during T helper cell type 1 (Th1-type) immune response pathways [40]. IDO could promote the proliferation of Tregs [41]. IDO can serve as a bridge of cross-talk between DCs and Treg cells, thus playing a role in maintaining immune homeostasis [42]. MSCs can secrete IDO and prostaglandin E2 [43], leading to the suppression of both T-cell and natural killer cell proliferation [44]. Of note, IDO produced by MSCs efficiently regulates innate and adaptive immunity [45]. The data indicate IDO as an important immune regulatory factor. Exosomes can carry IDO to extracellular to exert immunoregulatory effects [18]. Exosomes isolated from IDO-overexpressed MSCs produce immunosuppressive effects via increasing the number of Tregs in DCs [19]. In this research, accompanied by the acceleration of self-repair in IRI mice, MSCs-Exo-IDO administration also inhibited the produce of pro-inflammation factors and promoted the produce of anti-inflammation factor. Possibly, the alterations of inflammation factors were also related with the population of DCs and Tregs in kidney tissues. In addition, our results suggested that MSCs-exosome induced M2 polarization and M2-mediated anti-inflammation response, which effects were notably elevated by IDO overexpression in MSCs. That was the expression intensity of IDO carried by exosomes mainly responsible for the modulation of macrophages M2 polarization. These finding supports the counter-regulatory role of IDO on macrophages polarization, restraining excessive or inappropriate immune activation in tumor microenvironment [46] or interferon-*γ*-initiated inflammatory pathway [47]. Possibly, MSCs-exosomes-carried IDO promotes M2 polarization, exerting anti-inflammation effects.

## Conclusions

Our findings demonstrated that IDO-overexpressed MSCs-derived exosome promoted renal self-recovery after IR-induced AKI by the regulation of macrophages polarization. The repair ability of IDO-overexpressed MSCs-Exo was significantly higher than the parental MSCs-Exo. Possibly, IDO-based therapeutic strategy is an effective approach to elevate the improvement effects of MSCs-Exo on renal injury in AKI, leading to the delay from AKI to CKD progression.

## Supplementary Information

**Additional file 1: Fig. S1.** Identification of MSCs-Exo. **A** Western Blot was used to detect the expression level of exosome markers such as CD9, CD63 and CD81 in MSCs-Exo. **B** Morphology of MCS-Exo was observed under transmission electron microscope. Scale bar: 200 nm.

**Additional file 2: Fig. S2.** Identification of MSCs-Exo in kidney tissues. The isolated exosomes were labeled by DiD. On day 1 after injection of labeled exosome, the fluorescence intensity of DiD was monitored by IF staining in kidney tissues. Scale bar: 20 μm.

**Additional file 3: Fig. S3**. Effect of MSCs-Exo on that expression of M1 and M2 markers in macrophages. **A** Western Blot was used to detect the expression of iNOS and Arginase1 in LPS-induced RAW264.7 cells under the treatment of MSCs-Exo or not. **B** Quantization diagram of panel A as shown in the right.

**Additional file 4: Fig. S4**. Effects of IDO-targeted therapies on IRI mice. **A**-**B** Serum was obtained from vector plasmid-challenged sham mice, vector plasmid-challenged IRI mice, IDO-overexpressing plasmid-challenged IRI mice, and MSC-Exo-IDO-challenged mice on day 0, 1, 3 and 7 after IRI (n = 3/group at each time). Scr (**A**) and BUN (**B**) contents were detected by the corresponding ELISA kits. **C** Kidney tissues were collected from the four groups on day 3 after IRI (n = 3/group). Kidney tissues in different groups were obtained and histopathology was monitored by HE staining. Arrowheads indicated the damaged tubular. **D** Relative expression of IDO in kidney tissues as determined by qRT-PCR (n = 3/group). The line chart was presented by Means ± SD, and the statistical analysis was performed using one-way ANOVA. **P < 0.01.

## Data Availability

The data used to support the findings of this study are included within the article.

## Abbreviations

AKI: Acute kidney injury
I/R: Ischemia–reperfusion
IRI: Ischemia–reperfusion injury
CKD: Chronic kidney disease
ESRD: End-stage renal disease
TEC: Tubular epithelial cell
Scr: Creatinine
BUN: Blood urea nitrogen
HE: Hematoxylin–eosin
SDS-PAGE: Sodium dodecyl sulfate–polyacrylamide gel electrophoresis
PVDF: Polyvinylidene difluoride membranes
TNF-*α*: Tumor necrosis factor-alpha
