# Supplementary figures and images for: Exosome from indoleamine 2,3-dioxygenase-overexpressing bone marrow mesenchymal stem cells accelerates repair process of ischemia/reperfusion-induced acute kidney injury by regulating macrophages polarization
Source: Stem Cell Res Ther. 2022 Jul 28;13:367. doi: 10.1186/s13287-022-03075-9 (PMC9331485; doi:10.1186/s13287-022-03075-9)

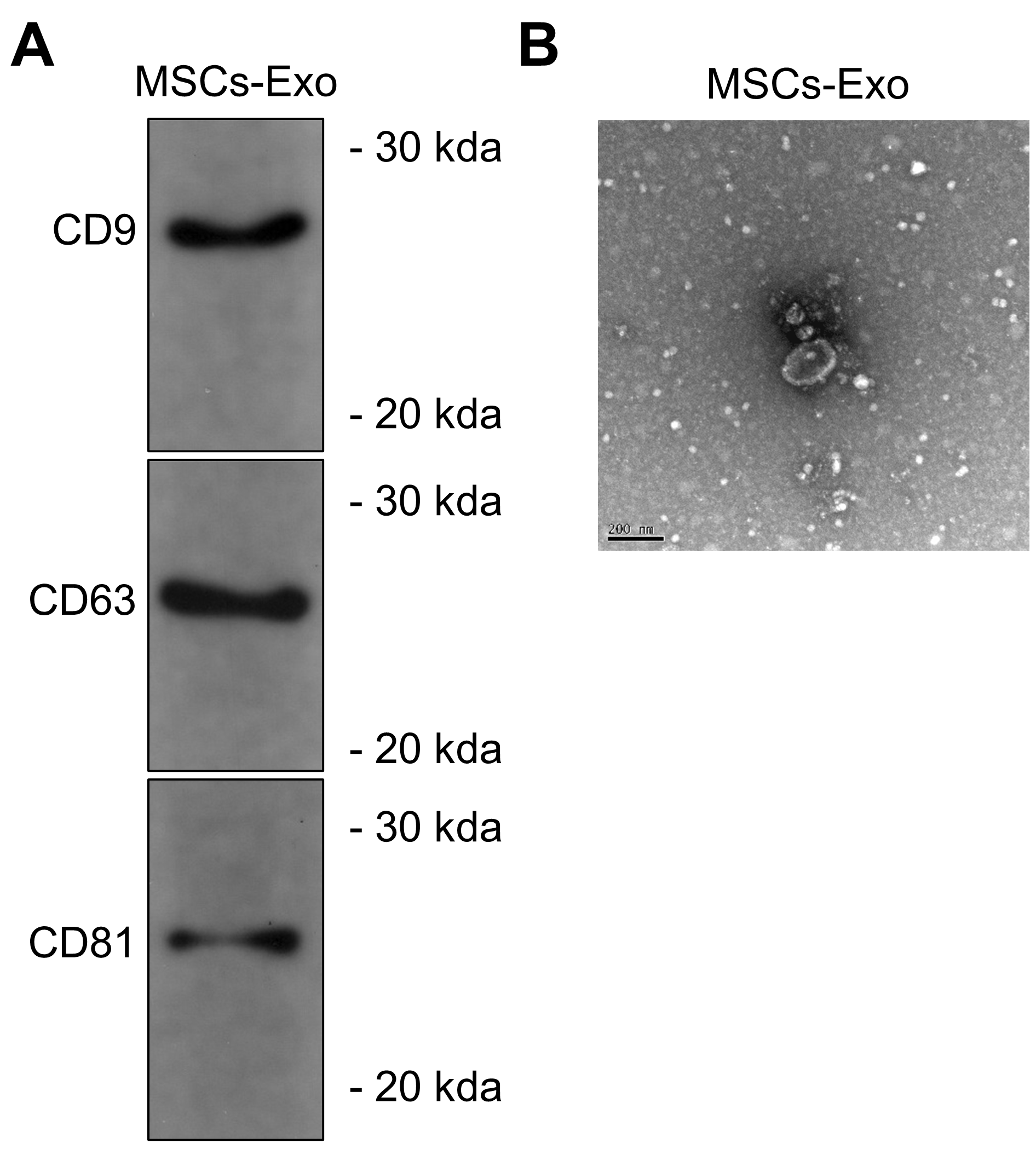

Supplement: Supplementary file 1 — Additional file 1: Fig. S1. Identification of MSCs-Exo. A Western Blot was used to detect the expression level of exosome markers such as CD9, CD63 and CD81 in MSCs-Exo. B Morphology of MCS-Exo was observed under transmission electron microscope. Scale bar: 200 nm. [file 13287_2022_3075_MOESM1_ESM.tif]

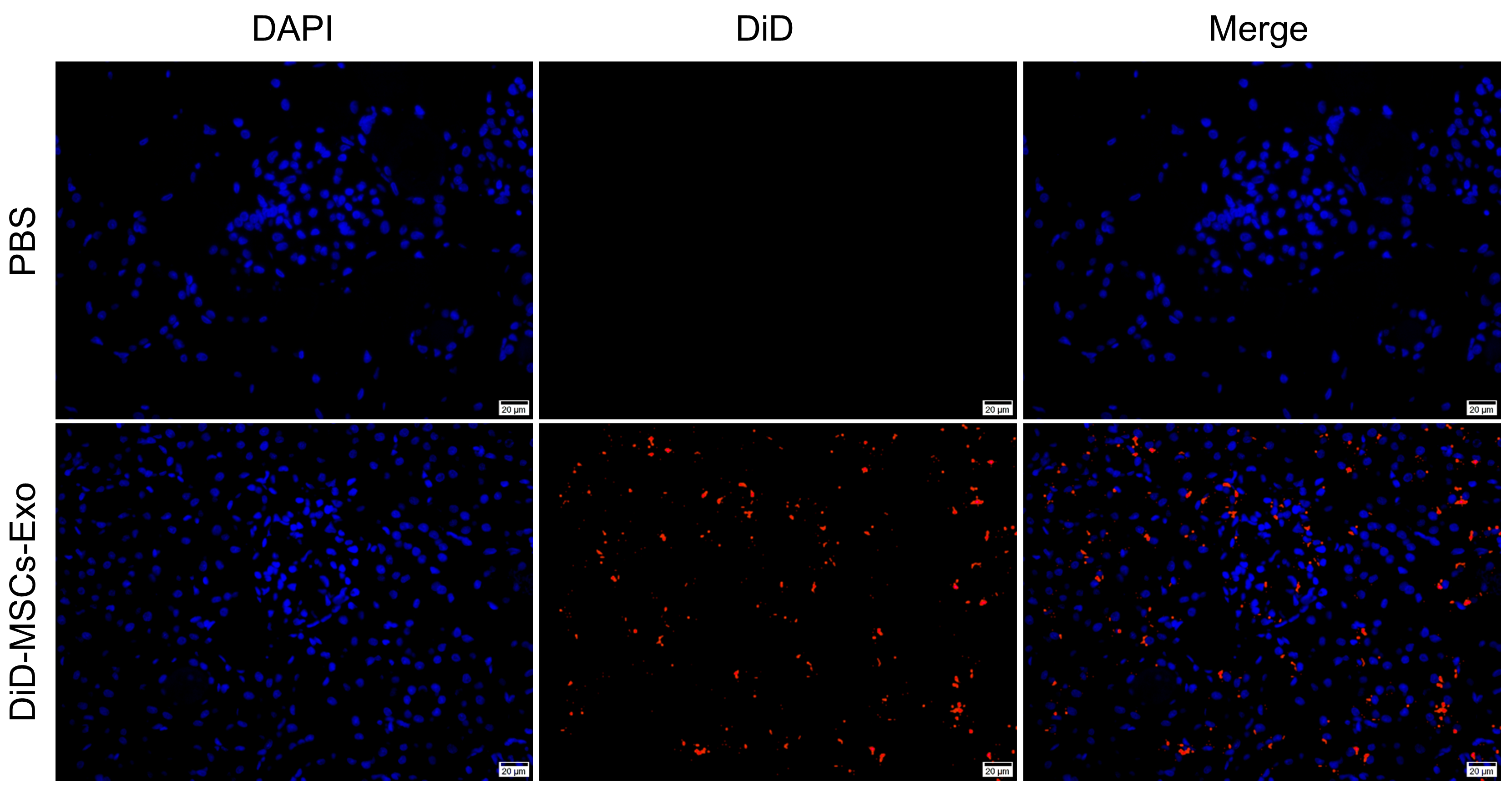

Supplement: Supplementary file 2 — Additional file 2: Fig. S2. Identification of MSCs-Exo in kidney tissues. The isolated exosomes were labeled by DiD. On day 1 after injection of labeled exosome, the fluorescence intensity of DiD was monitored by IF staining in kidney tissues. Scale bar: 20 μm. [file 13287_2022_3075_MOESM2_ESM.tif]

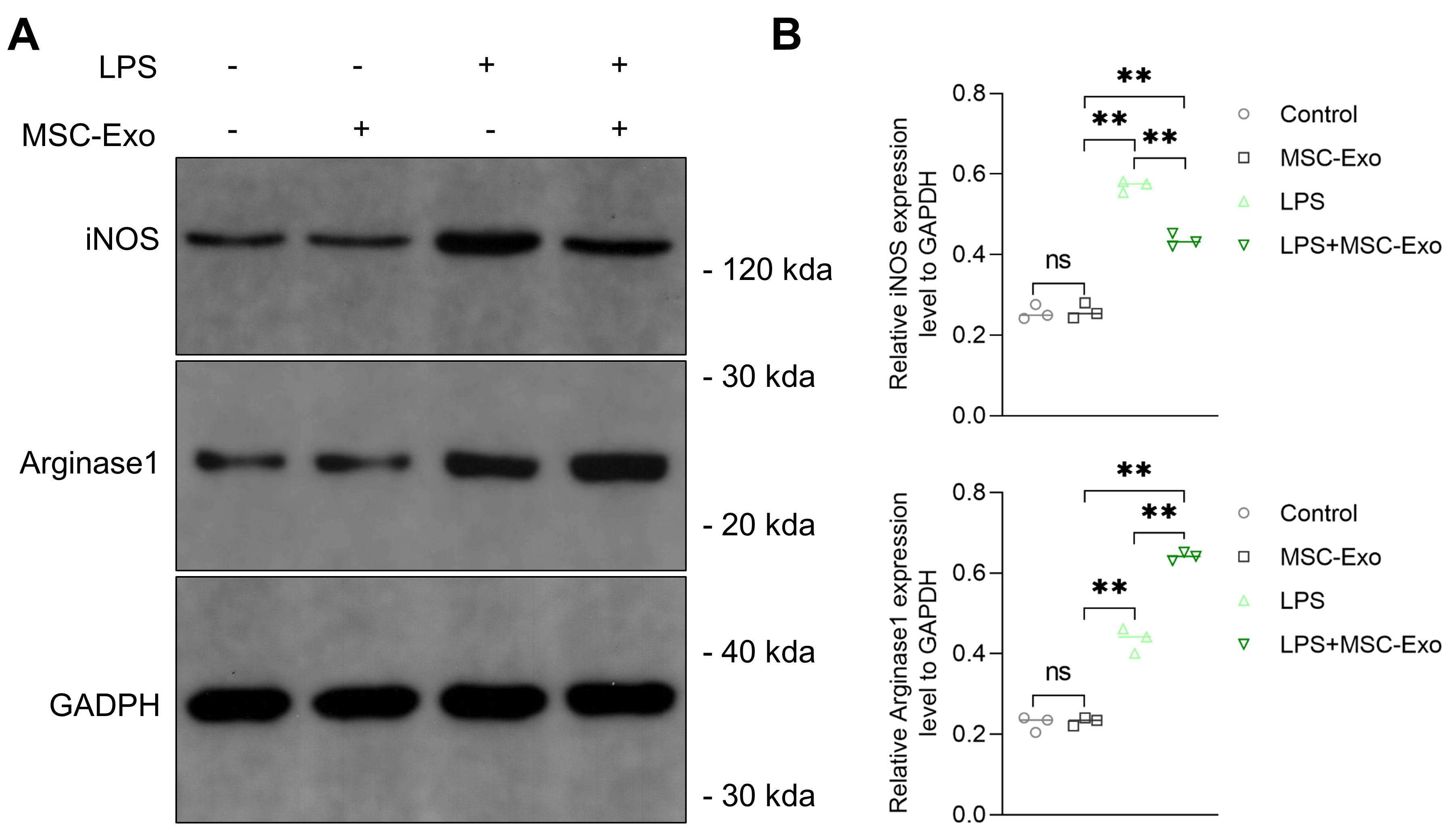

Supplement: Supplementary file 3 — Additional file 3: Fig. S3. Effect of MSCs-Exo on that expression of M1 and M2 markers in macrophages. A Western Blot was used to detect the expression of iNOS and Arginase1 in LPS-induced RAW264.7 cells under the treatment of MSCs-Exo or not. B Quantization diagram of panel A as shown in the right. [file 13287_2022_3075_MOESM3_ESM.tif]

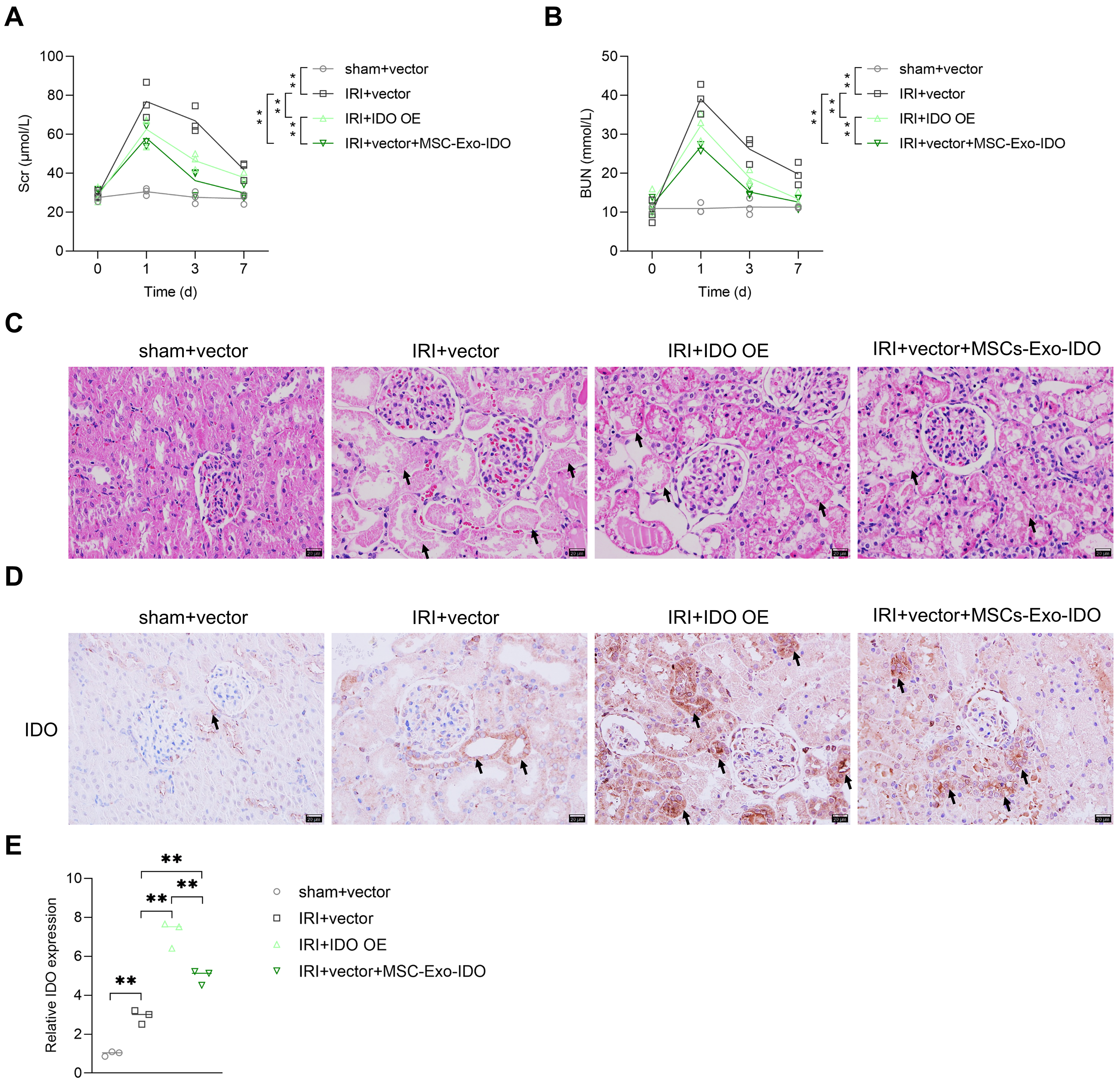

Supplement: Supplementary file 4 — Additional file 4: Fig. S4. Effects of IDO-targeted therapies on IRI mice. A-B Serum was obtained from vector plasmid-challenged sham mice, vector plasmid-challenged IRI mice, IDO-overexpressing plasmid-challenged IRI mice, and MSC-Exo-IDO-challenged mice on day 0, 1, 3 and 7 after IRI (n = 3/group at each time). Scr (A) and BUN (B) contents were detected by the corresponding ELISA kits. C Kidney tissues were collected from the four groups on day 3 after IRI (n = 3/group). Kidney tissues in different groups were obtained and histopathology was monitored by HE staining. Arrowheads indicated the damaged tubular. D Relative expression of IDO in kidney tissues as determined by qRT-PCR (n = 3/group). The line chart was presented by Means ± SD, and the statistical analysis was performed using one-way ANOVA. **P < 0.01. [file 13287_2022_3075_MOESM4_ESM.tif]
